# A Region Tracking-Based Vehicle Detection Algorithm in Nighttime Traffic Scenes

**DOI:** 10.3390/s131216474

**Published:** 2013-12-02

**Authors:** Jianqiang Wang, Xiaoyan Sun, Junbin Guo

**Affiliations:** 1 State Key Laboratory of Automotive Safety and Energy, Tsinghua University, Beijing 100084, China; 2 Suzhou INVO Automotive Electronics Co., Ltd., Suzhou 215200, China; E-Mail: sunxiaoyantsj@126.com; 3 Xi'an Institute of High-Tech, Xi'an 710025, China; E-Mail: gjb_202@163.com

**Keywords:** advanced driver assistance system, nighttime vehicle detection, vehicle taillights, pairing, tracking, time-series analysis model, adaptive thresholds

## Abstract

The preceding vehicles detection technique in nighttime traffic scenes is an important part of the advanced driver assistance system (ADAS). This paper proposes a region tracking-based vehicle detection algorithm via the image processing technique. First, the brightness of the taillights during nighttime is used as the typical feature, and we use the existing global detection algorithm to detect and pair the taillights. When the vehicle is detected, a time series analysis model is introduced to predict vehicle positions and the possible region (PR) of the vehicle in the next frame. Then, the vehicle is only detected in the PR. This could reduce the detection time and avoid the false pairing between the bright spots in the PR and the bright spots out of the PR. Additionally, we present a thresholds updating method to make the thresholds adaptive. Finally, experimental studies are provided to demonstrate the application and substantiate the superiority of the proposed algorithm. The results show that the proposed algorithm can simultaneously reduce both the false negative detection rate and the false positive detection rate.

## 1. Introduction

Traffic accident data indicates that nighttime accidents are more hazardous than those during the daytime [1], and that accidents caused by the rear-end collisions account for more than a third of all the accidents during nighttime [2]. In order to prevent these accidents, the rear-end collision warning system is an important part of the advanced driver assistance system (ADAS) [3]. With the rapid development of modern computer vision techniques, nighttime vehicle detection based on image processing techniques has been gained much attention in recent years.

During daytime, the typical features for vehicles detection include edge features, shape templates, shadows, bounding boxes of vehicles, etc. However, these features cannot be applied at nighttime, as the difference between the vehicles and the environment background is very low. At nighttime, the pair of taillights or headlights is the most commonly used feature to describe a vehicle [4–26]. For vehicle detection, the features, e.g., intensity, sizes, shape, texture, color, symmetry, are usually used to identify the pair of taillights at night.

Generally, detecting the pair of taillights includes main three steps: *i.e.*, bright spots segmentation, candidate taillights extraction, candidate taillights pairing. The candidate taillights are extracted by setting fixed thresholds of a series of features. However, the candidate taillights are disturbed by the traffic lights, mark lines, signs, *etc.* Additionally the road environments are harsh due to the braking, lane-changing, camera dithering, *etc.* Thus, vehicles detection using fixed values is not satisfactory.

To improve the accuracy of taillights detection, current research is focused on the following two aspects: the first aspect is the use of shape descriptors to represent the taillights and utilize the Support Vector Machine (SVM) classifier to train the historical taillights data [4]. Similar works can be found in [5,6]. This method could improve the detection rate effectively, but the extraction rule is also fixed in essence and the inter-frame information is not fully used.

Another aspect is adding a tracking algorithm to taillights detection to use the inter-frame information. A classic work is proposed by O'Malley *et al.* [7,8], who used the Kalman filtering method to track the location of the taillights by the previous location. Then, when the taillights detection is missing, the estimated location is used to compensate for the unavailable detection. Following O'Malley *et al.* [7,8], many variants and extensions have been reported for taillights detection [5,9,10]. Similar ideas can also be found in [11,12], where the templates of specific rules for taillights detection are tracked. This tracking method can be further categorized into two types: tracking the pair of taillights [7,8,13,14] and tracking the taillight spots [6,9,15]. These tracking methods can effectively reduce vehicle detection false negative rates, but it is difficult to reduce the false positive detection rate. Moreover, the thresholds for extracting the taillight spots are fixed for all frames.

From the above review of related researches, we observe that there are two issues remaining to be resolved. The first is to improve the detection accuracy. The second is to make the feature thresholds adaptive. In order to solve these issues, this paper proposes a new region tracking-based detection algorithm based on the time series analysis model [27]. Firstly, a time series analysis model is introduced to predict vehicle positions and the possible region (PR). Then, in the next frame the vehicle is only detected in the PR, which is much narrower than the whole image. Therefore, the detection time is reduced and the false pairing between the bright spots in the PR and the bright spots out of the PR is eliminated. This is the main contribution of the paper, which is not fully explored before. Moreover, the feature thresholds are adapted based on the similar features of the bright spots in the PR in the previous frame. This is another contribution of the paper since the detection rate is improved. Finally, some experiments based on the practical video data taken at night are provided to substantiate the superiority of the proposed method compared with the existing classic methods in the literature.

The remaining parts are organized as follows: Section 2 introduces the general vehicle detection process based on the taillight pairs in the global image. In Section 3, we present the region tracking based detection algorithm at nighttime and the method to make the rule thresholds adaptive. Section 4 presents the experimental results to illustrate the application and usefulness of the developed algorithm. Section 5 concludes this study with a discussion.

## 2. Global Rule Based Vehicle Detection

In this section, we briefly introduce the global rule based vehicle detection algorithm. The global algorithm includes three major steps, *i.e.*, bright spots segmentation, candidate taillights extraction and candidate taillights pairing.

### 2.1. Bright Spots Segmentation

The improved Otsu method based on the cumulative histogram presented in [28] is applied for the bright spots segmentation in this paper. As the improved Otsu method is presented in Chinese, we summarize the improved Otsu method as follows: the classic Otsu method assumes that the image contains two classes of pixels or histogram (e.g., foreground and background), and calculates the optimum threshold separating the two classes so that their intra-class variance is minimal [29]. However, the taillights are bright and tiny, and thus the pixels of taillights take up a small portion in the whole image. Then, the traditional Otsu method could not segment the bright spots out of the image. To solve this problem, the improved Otsu method is suggested.

**Figure 1.**
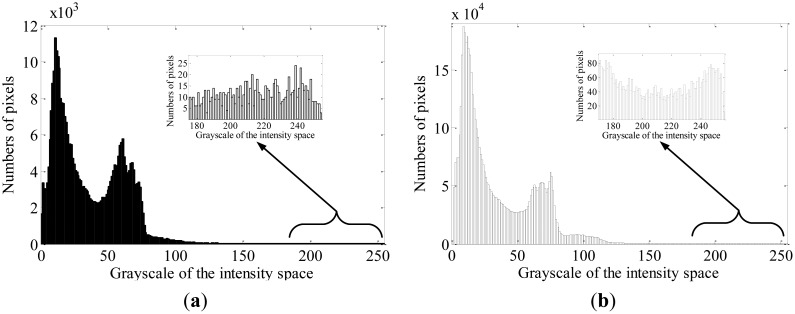
(**a**) Intensity histogram of a single frame; (**b**) Cumulative intensity histogram of consecutive fifteen frames.

When the camera is installed in host vehicle, the region where the preceding vehicles are most commonly locating is usually taken as the region of interest (ROI). Then, the detection is only performed in the ROI. In this paper, we regard the [200–400] pixels region in the y-axis direction as the ROI. For saving the computation in bright spots extraction, the color image should be transformed into a gray image. We regard the intensity component as the transformed gray image due to the stable brightness of the taillights. The intensity histogram of a single frame and the cumulative intensity histogram of consecutive fifteen frames are respectively shown in Figure 1 where the cumulative brightness histogram is calculated by Equation (1):

(1) $$\{\begin{array}{cc} \begin{array}{l} H(k)=n_{k}\\ L(t,k)=\sum_{i=1}^{t}H_{i}(k) \end{array} & \text{for k}=0,1,\cdots ,l-1 \end{array}$$

In Equation (1), *k* denotes the gray level, *l* is the total number of gray values in the histogram, *t* is the number of cumulated images which could be chosen as other values, *n_k_* denotes the number of gray level *k*, and *L*(*t*, *k*) denotes the cumulative number of gray level *k* based on the *t* cumulative images.

**Figure 2.**
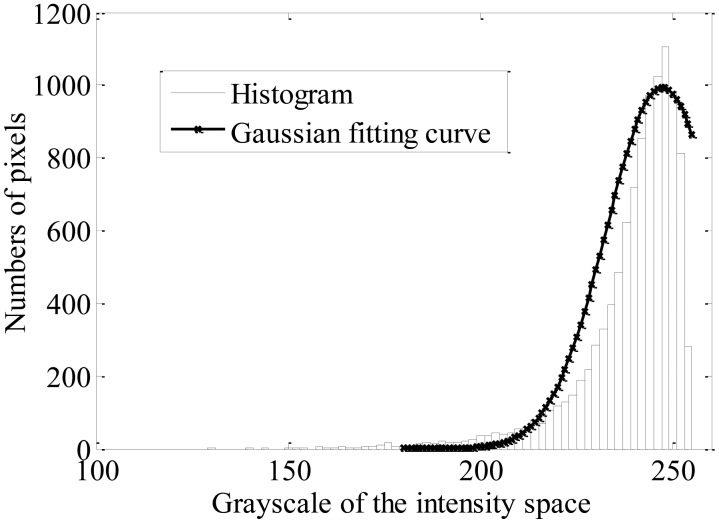
Histogram illustrating the brightness distribution of taillight pixels.

It can be observed that there is no special feature in the higher gray level interval in Figure 1a, while there is an obvious bimodal distribution in the corresponding interval in Figure 1b. The result shows that there is an obvious difference between the targets and the background in the cumulative histogram, which satisfies the application condition of the Otsu method. Moreover, as the taillight spots are concentrated in the brightest region, the segmentation should be implemented in the brighter region from a lower gray value to 255. First, a statistical segmentation threshold *T_s_* is derived from the distribution of the taillights region in brightness histogram for a database of 300 images, which are captured in different traffic scenes. A Gaussian curve is fitted to the brightness histogram data, as shown in Figure 2, and the statistical threshold *T_s_* is obtained as 214 at the probability point (*μ* − 2*σ*). By assuming that the statistical threshold *T_s_* is the ideal threshold for segmentation, the initial segmentation threshold *T_I_* can be computed by:

(2) $$T_{s}=(T_{I}+l-1)/2$$

**Figure 3.**
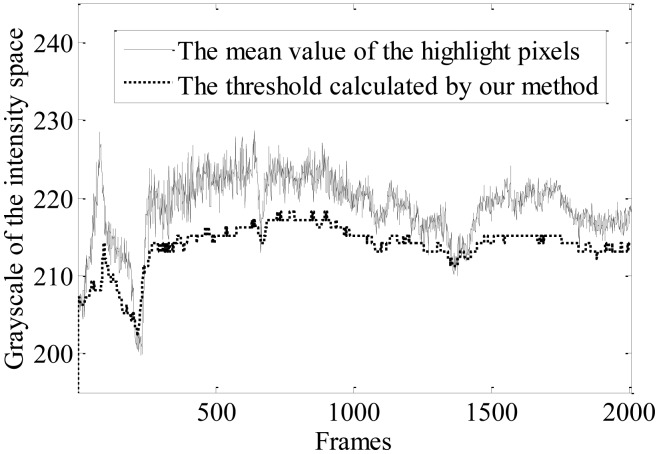
Tendency of the adaptive threshold.

Thus:

(3) $$T_{I}=T_{S}\times 2-(l-1)$$

where *l* = 256 is the total number of gray levels. Therefore, we obtain that *T_I_* = 173. Then, the interval from 173 to 255 of the cumulative histogram is utilized for segmentation by the Otsu method. The optimal segmentation thresholds calculated by the improved Otsu method for 2,000 frames are presented in Figure 3. It can be seen that the variation of the thresholds agrees with the mean value of highlight pixels, the gray of which are greater than 150. This implies that the improved Otsu method is adaptive to the variation of the luminance of the traffic scenes.

### 2.2. Candidate Taillights Extraction

To extract the candidate taillights from the bright spots, the connected-component extraction technique [18] is performed to locate the bright spots. The extracted connected-components include the bright spots of other disturbed sources, which also exist in nighttime traffic scenes, such as street lamps, traffic lights and road reflector plates, *etc.* To extract the taillights out of the disturbed spots, the rules are based on the features of the area, shape, color and size. Let *C_i_* (*i* = 1, 2, ⋯, *p*) denote the *i*-th connected-component of the current frame, and *B_i_* denote the bounding box which encloses *C_i_*; the rule-based taillights extraction process is summarized as follows. If a candidate bright spot is considered as the candidate taillight, the following rules should be satisfied. In our system, the requirement of detection distance from host vehicle is 60 m. The selection of the thresholds should satisfy this requirement.

(1) The area of the candidate bright spots should satisfy that:
  (4) $$A(C_{i})\geq TH_{A}$$
  where *A*(*C_i_*) is the area of the *i*-th bright spot, and *TH_A_* is the threshold of area.
(2) The typical characteristic of the taillights is the redness at nighttime. The red level of the bright component is computed by:
  (5) $$R(C_{i})=\frac{\sum_{j\in C_{i}}g_{j}+b_{j}}{2\sum_{j\in C_{i}}r_{j}}$$
  where *R*(*C_i_*) is the red level of the *i*-th bright component, *r_j_*, *g_j_*, and *b_j_* denote the gray value of *j*-th pixel in the *R*, *G*, and *B* channels of the candidate bright spot. In general, the average value of red level in actual taillights is less than 1, and the smaller the average value of red level is, the more redness of the component is. Therefore, we use this typical characteristic of taillights to distinguish them from other bright spots, that is:
  (6) $$R(C_{i})\leq TH_{R}$$
  where *TH_R_* is the threshold of the red level.
(3) The enclosing bounding box of the candidate taillights must satisfy the required shape of the actual taillight. Let *W*(*B_i_*) and *H*(*B_i_*) denote the width and height of the bounding boxes, respectively; then the width *W* and the aspect ratio of the enclosing box of the candidate bright spots must satisfy the following constraints, respectively:
  (7) $$\{\begin{array}{l} W(B_{i})\geq TH_{W}\\ TH_{\text{WHR}1}\leq W(B_{i})/H(B_{i})\leq TH_{\text{WHR}2} \end{array}$$
  where the thresholds *TH_WHR_*_1_ and *TH_WHR_*_2_ are selected to suitably determine the shape of a potential taillight, and *TH_W_* is the threshold of the width of the bounding box.

Like the existing vehicle detection algorithm, these thresholds of the taillights extraction rules are fixed based on experience or the statistical images.

### 2.3. Candidate Taillights Pairing

To determine the position of target vehicles in road scenes, the pairing method is used to cluster potential taillights in pairs. Although the shape of the vehicle taillights may be irregular, the taillights in one vehicle are symmetrical and placed in pairs. The symmetry of a pair of taillights can be described by the difference of area, the difference of the vertical coordinate value of the two spots, and the correlation between the two taillights' regions. The correlation of the taillights' regions in pairs can be described by the region self-correlation in the enclosing box of taillights and measured by the cross-correlation function [7,8]. Generally speaking, the aspect ratio (width/height) of a vehicle is approximated to be 2.0 [30,31]. Therefore, the taillights pairing method is based on the above features and the corresponding criterions are given as follows:

(1) First, the candidate taillights are pairing according to the area and the vertical coordinate value. The following should be satisfied:
  (8) $$\text{abs}(A(C_{i})-A(C_{j}))\leq THd_{A}$$
  (9) $$\text{abs}(C_{Y}(C_{i})-C_{Y}(C_{j}))\leq THd_{C_{Y}}$$
  where *A*(·) is the area of the candidate taillight to be paired, *C_Y_*(·) is the vertical coordinate value of the candidate taillights' centroid, and *THd_A_*, *THd_CY_* are the thresholds to *A*(·), *C_Y_*(·), respectively.
(2) Let *u*(*B_i_*), *d*(*B_i_*), *l*(*B_i_*), and *r*(*B_i_*) be the top, bottom, left and right coordinates of the enclosing bounding box *B_i_*, respectively; then the enclosing bounding box of the candidate taillight must satisfy the following condition:
  (10) $$TH_{W1}\leq \max(r(B_{i}),r(B_{j}))-\min(l(B_{i}),l(B_{j}))\leq TH_{W2}$$
  (11) $$TH_{\text{Ratio}1}\leq \frac{\max(r(B_{i}),r(B_{j}))-\min(l(B_{i}),l(B_{j}))}{\max(d(B_{i}),d(B_{j}))-\min(u(B_{i}),u(B_{j}))}\leq TH_{\text{Ratio}2}$$
  where *TH_W_*_1_ and *TH_W_*_2_ are utilized to reflect the width of the paired vehicle taillights, and *TH_Rotio_*_1_ and *TH_Rotio_*_2_ are utilized to reflect the rectangular shaped appearance of the paired vehicle taillights.
(3) The taillights belong to the same vehicle usually have a high correlation value, as they have similar size, shape, and luminance values. Therefore, the higher correlation value is, the higher possibility that the spots belong to the same vehicle. Then, the following should be satisfied:
  (12) $$\text{Correlation}(B_{i},B_{j})\geq TH_{\text{corr}}$$
  where the threshold *TH_corr_* is used to limit the correlation value of the candidate pair of taillights.

The thresholds for pairing candidate taillights in Equations (8)–(12) are also fixed values determined by experience or the statistical images. If there is an overlapped spot between two taillight pairs, the pair with smaller difference and higher degree of symmetry is selected to describe a vehicle.

## 3. The Region Tracking-Based Vehicle Detection Algorithm

As discussed above, the rules of extracting and pairing taillights in the existing vehicle detection algorithm are usually limited by fixed thresholds, which cannot be adapted to real traffic scenes. Moreover, adding the tracking algorithm can only effectively reduce the false positive detection rate, but cannot reduce the false negative detection rate. Therefore, we propose a region tracking-based vehicle detection algorithm in this section. First, the potential vehicle is detected by the global rule-based algorithm. Once the pair of the taillights is confirmed as a vehicle, a time series analysis model is applied to predict the position of the target vehicle. Then, the possible region (PR) of the vehicle is constituted by the predicted position. Additionally, the vehicle is only detected in the PR and the tracker is updated with the detection result. If vehicle detection is only implemented in the PR, a new vehicle coming into the visual field would be missed. Therefore, it is necessary to identify the new vehicle in the remaining area of the image at a frequency. Combining the global detection algorithm with the region tracking-based vehicle detection algorithm could improve the accuracy of detection rates. In the following, we first introduce the flow diagram of the presented algorithm.

### 3.1. The Flow Diagram

**Figure 4.**
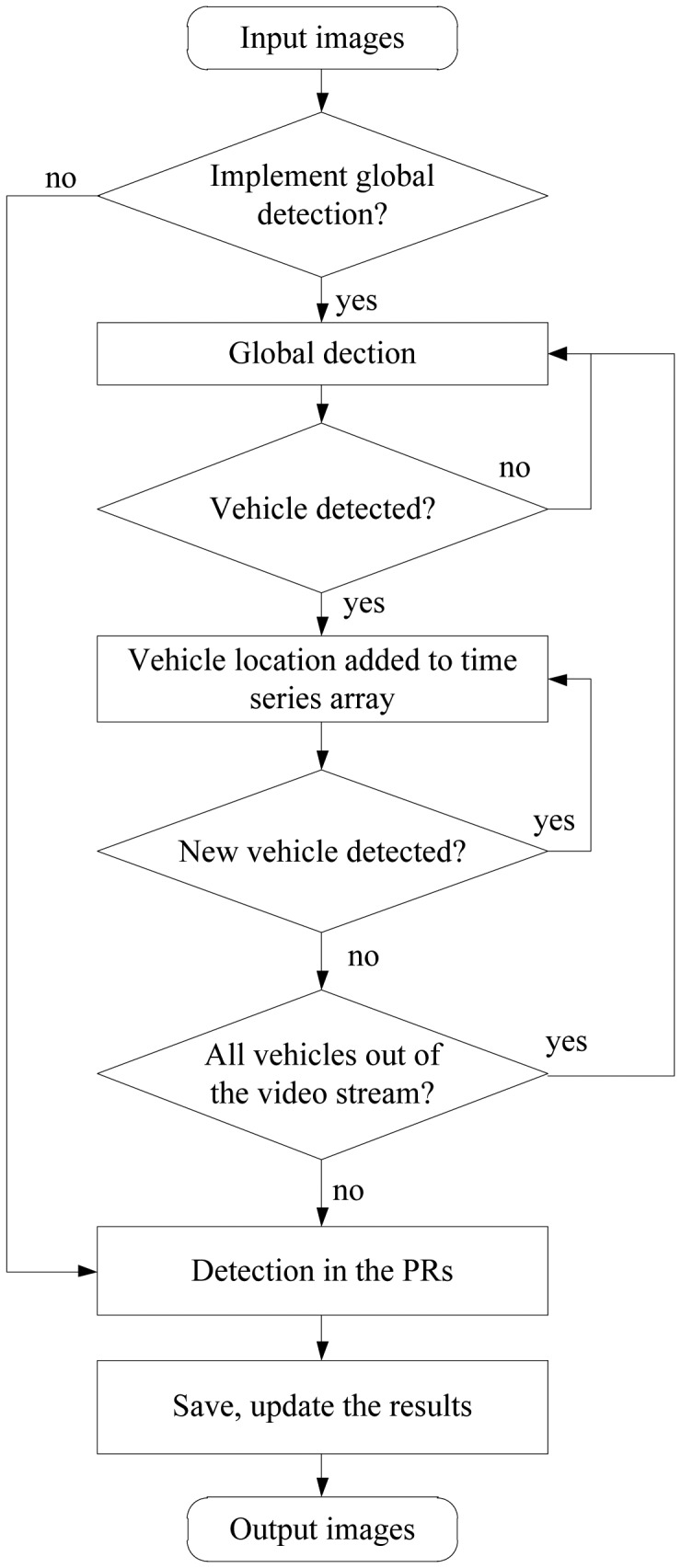
The flow diagram.

The flow diagram of the region tracking based vehicle detection algorithm is shown in Figure 4. If the algorithm is used directly, two problems exist. First, when a target vehicle has moved out of the video stream, the algorithm will keep detecting the target vehicle in the PR and this may lead to unintended results. Therefore, the removed vehicle should be dynamically removed from the tracker list. Second, new target vehicles will appear in the video stream randomly in actual traffic scenes, so they should be added in a new tracker to avoid negative detection.

The two problems are addressed as follows. For the first problem, if a vehicle remains undetected in five successive frames, it would be regarded as a disappearing target, and the corresponding tracker would be removed from the time series arrays. For the second problem, if a vehicle keeps being detected in five successive frames, it indicates that a target vehicle has appeared. For the case where a vehicle is already being tracked, the remaining region after eliminating the regions where stable vehicles exist is used to search whether a new target vehicle appears in every twenty frames. The newly detected vehicle would be added to the time series arrays for tracking. Moreover, if all vehicles are disappearing in the video stream, the global detection algorithm will be used and the time series arrays would be initialized.

**Figure 5.**
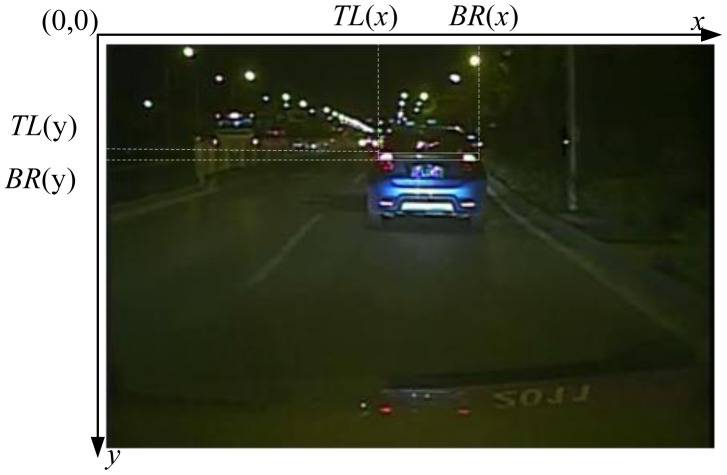
Vehicle position mark.

The four parameters of the bounding box surrounding the pair of the taillights (*i.e.*, *x*-position and *y*-position of the top-left (*TL*) coordinates, *x*-position and *y*-position of the bottom-right (*BR*) coordinates) are regarded as the random tracking time series data to predict the position of target vehicles in the next frame image, as shown in Figure 5. It can be observed that the position of a target vehicle is determined by the top-left and bottom-right points. Therefore, the *x*-coordinate and *y*-coordinate of point *TL* and point *BR* are used to describe the position of the target vehicle in the two directions, respectively. Let *L* = [*TL*(*x*) *BR* (*x*) *TL*(*y*) *BR*(*y*)] represent the position of a target vehicle; then the target position at time *t* can be expressed as *L_t_* = [*TL*(*x_t_*) *BR*(*x_t_*) *TL*(*y_t_*) *BR*(*y_t_*)], and the target position at the time series *t*_1_, *t*_2_,…, *t_k_* can be expressed as [*L_t_*_1_, *L_t_*_2_,…, *L_tk_*].

The proposed approach in this paper is composed of three main steps as follows:

Step 1: Initialize the time series arrays of the tracked vehicles' position
  As there is no original position data in the time series arrays for tracking, the starting five frames of video stream should be detected by applying the global detection algorithm. The process to detect the five frames costs the same time as the existing vehicle detection algorithms.
Step 2: Predict the PRs
  The detected vehicles are tracked by the time series analysis model. The PR can be extended based on the rectangle [*TL*(*x_t_*) *BR*(*x_t_*) *TL*(*y_t_*) *BR*(*y_t_*)]. In this paper, the PR is obtained by extending the rectangle [*TL*(*x_t_*) *BR*(*x_t_*) *TL*(*y_t_*) *BR*(*y_t_*)] outwards with 5 pixels.
Step 3: Detect the vehicle in the Predicted PRs
  The global detection algorithm is implemented only in the PR, if the pair of the taillights is verified as a vehicle. Moreover, the thresholds for the detection in the PR are also updated, which is presented in Section 3.3. Additionally, if a vehicle cannot be detected at time *t*, the predicted position via the AR model is utilized to compensate it and it is considered as the true position. This could avoid the missing detection rate.

### 3.2. Tracking Method Based on AR Model

Tracking the detected vehicles is performed for two main reasons, *i.e.*, to predict the position of vehicles and identify the PR region to be detected in the next frame, and to extrapolate features (size and position) of the vehicle if the detection failed in a short period. As the Kalman filtering method has to know the movement model of the vehicle and only adapts to the linear model, and the particle filter method has poor real-time properties, we use the time series model to track the vehicles.

Time series analysis is a statistical method for dynamic data processing. It can abstract the data variation law and predict the developmental trend by analyzing the prior data [27]. This method has considered the dependence of the observed data in time sequence and the randomness of the unnecessary factors. The dependence and randomness of data are analyzed by a stochastic dynamic model.

The most common examples of time series models are the auto-regression model (AR), moving average model (MA) and autoregressive moving average model (ARMA). The ARMA model is described by the following:

(13) $$x_{t}-(\varphi_{1}x_{t-1}+\varphi_{2}x_{t-2}+\cdots +\varphi_{p}x_{t-p})=\mu_{t}-\theta_{1}x_{t-1}-\theta_{2}x_{t-2}-\cdots -\theta_{q}x_{t-q}$$

and the error vector is given by:

(14) $$\mu_{t}={\bar{x}}_{t}-x_{t}$$

where *x_t_* is the state vector; *μ_t_* is the error vector derived from the difference of the measurement vector *x̅_t_* and the state vector *x_t_* for a target at time *t; φ_i_* (*i* = 1, 2,…, *p*) are the autoregressive parameters, which are used to define the linear relationship between the current motion state and the prior motion states from the time *t*-*p* to the time *t*-1; *θ_i_* (*i* = 1, 2,…, *p*) are the moving average parameters, which are used to define the linear relationship between the error term of current motion state and the prior motion states from the time *t*-*p* to the time *t*-1. Equation (13) is the autoregressive moving average model ARMA (*p*,*q*). The left part is the auto-regression model AR (*p*), and the right part is the moving average model MA (*q*). It can be seen from Equation (13) that the part of MA (*q*) contains the residual term, which must be calculated by the current measurement value.

However, the current measurement value cannot be acquired in the prediction process. It needs a large amount of calculation to get the parameters of the MA (*q*) model by solving the nonlinear equation. As the purpose of the tracking algorithm in this paper is only to detect the PR and compensate the missing detections, the requirement is easy to satisfy. Thus, the AR model is chosen as the tracking model in this paper.

For the time series {*x_t_*}, *t* = 1, 2,…, *N*, the AR model can be described by:

(15) $$x_{t}=\varphi_{1}x_{t-1}+\varphi_{2}x_{t-2}+\cdots +\varphi_{p}x_{t-p}+\mu_{t}$$

and the measurement error vector is given by:

(16) $$\mu_{t}={\bar{x}}_{t}-x_{t}$$

where the error vector *μ_t_* is normally and independently distributed, *i.e.*, *μ_t_* ∼*NID*(0, *φ*^2^*_μ_*), *φ*^2^*_μ_* can be calculated by the following equation:

(17) $$\varphi_{\mu }^{2}=\frac{1}{N-p}{\sum_{t=p+1}^{N}\left(x_{t}-\sum_{i=1}^{p}\varphi_{i}x_{t-1}\right)}^{2}$$

From Equation (17), it can be seen that *φ*^2^*_μ_* is expressed by *φ_i_*. It indicates that *φ_i_* (*i* = 1, 2,…, *p*) could be estimated by the least square estimation. The estimated parameters are unbiased and highly precise and the estimation process can be expressed by the following equation:

(18) Y=Xφ+μ

where:

(19) $$\{\begin{array}{l} Y={\left[x_{p+1}\;x_{p+2}\;\cdots \;x_{N}\right]}^{\text{T}}\\ \varphi ={\left[\varphi_{1}\;\varphi_{2}\;\cdots \;\varphi_{p}\right]}^{\text{T}}\\ X=\left[\begin{array}{lll} x_{p} & x_{p-1} & \ldots x_{1}\\ x_{p+1} & x_{p+2} & \ldots x_{2}\\ x_{N-1} & x_{N-2} & \ldots x_{N-p} \end{array}\right]\\ \mu ={\left[\mu_{p+1}\;\mu_{p+2}\;\cdots \;\mu_{N}\right]}^{\text{T}} \end{array}$$

Thus, the estimation results is given by:

(20) $$\varphi ={\left(X^{T}X\right)}^{-1}X^{T}Y$$

Since the position of the target vehicle is described by the *x*-coordinate and *y*-coordinate of point *TL* and point *BR* and these four coordinates mentioned above may have different motion trends, the vector [*L_t_*_1_, *L_t_*_2_,…, *L_tk_*] is split into four one-dimensional time series for prediction. Then, the AR model is utilized to track the motion trend of the target vehicle in image sequences. However, with the rising order of the AR model, computational complexity would arise and the detection speed would be slow. Based on the above analysis, we use the AR model of order 3 to predict the position of target vehicles, as in [27].

**Figure 6.**
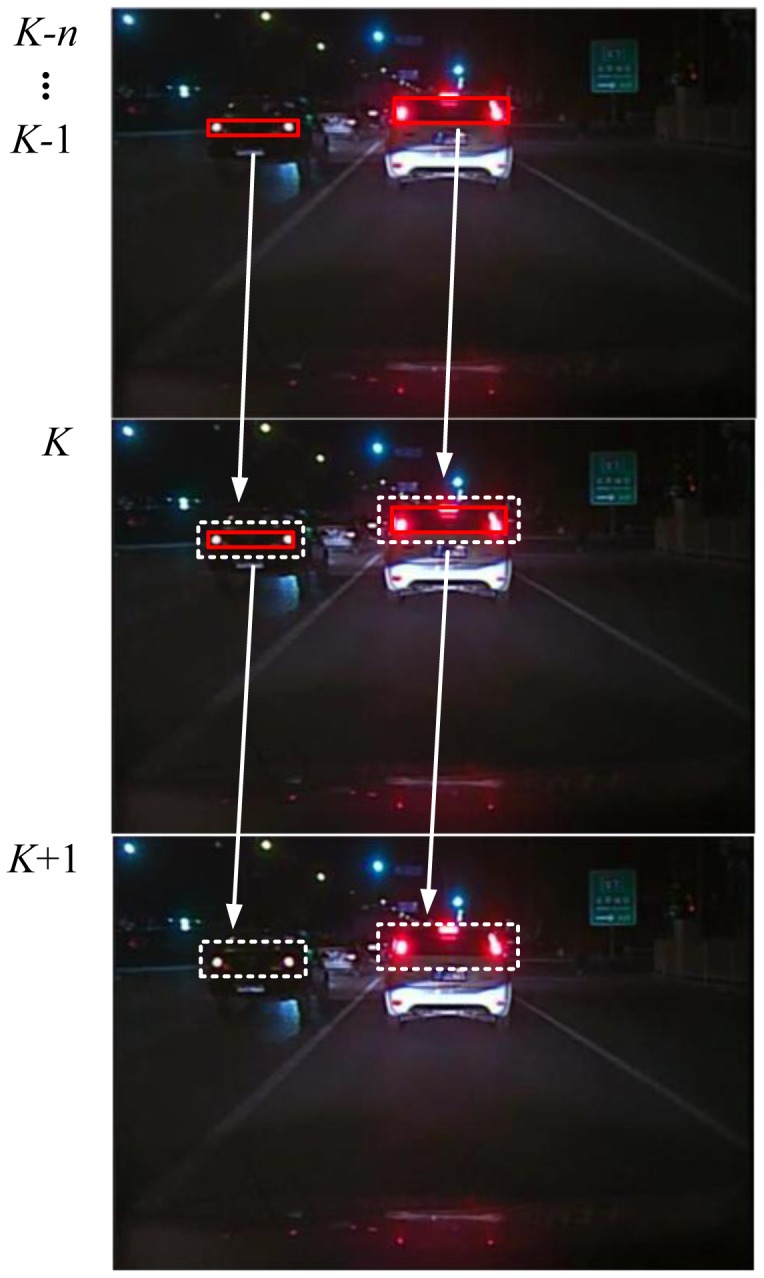
Illustration of the tracking-based detection algorithm.

Figure 6 shows the vehicle detection process of the proposed method. The positions of the detected vehicles obtained by the global based detection algorithm of the previous five frames are added to the time series arrays to predict the vehicles' position in the next frame. The predicted vehicle positions are extended by five pixels outwards to get the PRs in the next frame, as shown in the second picture of Figure 6. The target vehicle in each PR is detected by the improved detection algorithm as described in Section 3.3. Finally, the detection result is added to the dynamic time series array, so the model could continue to predict the vehicle position in the subsequent frames.

### 3.3. PR-Based Detection Rules

In this subsection, we propose a PR-based detection algorithm. For the global based algorithm, if the range of the fixed thresholds is set broad enough to detect more brightness spots, then more interfering brightness spots would be detected too. However, on the contrary, if the range of the fixed thresholds is set too narrow to limit the disturbed bright spots, many taillight spots are also restricted. Therefore, it is difficult or even impossible for the fixed threshold to simultaneously minimize the disturbances and the missing taillights (*i.e.*, false detection rate and false negative rate). However, the proposed tracking- based detection algorithm provides a practical way to solve this problem.

Although the characteristics of the taillight spots are affected by the taillights type, the braking operation of the target vehicle, and the distance between the host vehicle and the target vehicle, the taillight pairs of the same vehicle between successive frames have very similar features. Based on this principle, we present an adaptive thresholds updating method. The features chosen for the taillights extraction and pairing are the same as the global detection algorithm, *i.e.*, the size, red level, width, ratio of width and height of bounding box, the brightness, and the correlation of the potential taillights region, *etc.* The adaptive thresholds updating method is presented in the following equations, where *L*_1_ and *L*_2_ represent the left and right taillight in the previous frame, respectively.

(21) $$TH_{A}=\max(3,0.4\times \min(A(L_{1}),A(L_{2})))$$

(22) $$TH_{R}=\max(R(L_{1}),R(L_{2}))-0.2$$

(23) $$TH_{w}=\min(\max(W(L_{1})-2,3),\max(W(L_{2})-2,3))$$

(24) $$\{\begin{array}{c} TH_{\text{WHR}1}=\min(W(L_{1})/H(L_{1}),W(L_{2})/H(L_{2}))-0.5\\ TH_{\text{WHR}2}=\min(W(L_{1})/H(L_{1}),W(L_{2})/H(L_{2}))+0.5 \end{array}$$

(25) $$THd_{A}=\max(6\times \text{abs}(A(L_{1})-A(L_{2})),1)$$

(26) $$THd_{c_{Y}}=\text{abs}(C_{Y}(L_{1})-C_{Y}(L_{2}))+5$$

(27) $$\{\begin{array}{c} TH_{W1}=\max(r(L_{1}),r(L_{2}))-\min(l(L_{1}),l(L_{2}))-5\\ TH_{W2}=\max(r(L_{1}),r(L_{2}))-\min(l(L_{1}),l(L_{2}))+5 \end{array}$$

(28) $$\{\begin{array}{c} TH_{\text{Ratio}1}=0.5\times (\frac{\max(r(L_{1}),r(L_{2}))-\min(l(L_{1}),l(L_{2}))}{\max(d(L_{1}),d(L_{2}))-\min(u(L_{1}),u(L_{2}))})\\ TH_{\text{Ratio}2}=2.5\times (\frac{\max(r(L_{1}),r(L_{2}))-\min(l(L_{1}),l(L_{2}))}{\max(d(L_{1}),d(L_{2}))-\min(u(L_{1}),u(L_{2}))}) \end{array}$$

(29) $$TH_{\text{corr}}=\text{Correlation}(L_{1},L_{2})-0.2$$

From the above equations, it can be observed that the thresholds in this frame are mainly determined by the previous frame. The thresholds are extended to an extensive range based on the values of the previous frame. There are two advantages of the proposed tracking-based detection algorithm for implementing the PR-based detection rules. The first is that there is only one vehicle in the tracked rectangle, thus the thresholds can be centralized for a specific vehicle. In other words, the thresholds of one vehicle are only determined by the feature values of this vehicle in the previous frame. The second is that the rectangle is relatively small, the number of the disturbed bright spots is very small and thus a high detection rate can be derived.

Compared with the global detection algorithm, the detection thresholds of the PR-based detection algorithm are adapted to real-time changes of the target vehicle, thus the detection rate increases. Moreover, as the PR-based detection algorithm is only implemented in the PR, false detections can be mostly avoided. In summary, the proposed algorithm provides a practical way to simultaneously minimize the disturbances and the missing taillights (*i.e.*, false detection rate and false negative rate).

## 4. Experimental Studies

In this section, we provide some experimental studies to compare the performance of the proposed algorithm with some classical works existing in the literature. As the offline implementation of the detection algorithm through a PC can relatively reflect the performance of the algorithm [3,14,20,21,25], we make the comparison using MATLAB R2009b on a Pentium Dual Processor −2.70 GHz personal computer platform with 2 GB memory for illustrative purposes. The vision system for acquiring the road environment video stream is mounted behind the windshield of the camera-assisted car. The frame rate of the vision system is 30 frames per second and the size of the grabbed image sequence is 720 × 480 pixels with 32 bit true color. Traffic situations in urban roads are categorized as urban motorways and urban arterial roads. Videos captured in urban roads include various traffic situations such as the proceeding car cutting into our lane, the host car cutting into another lane and driving on curvy roads, *etc.* There are a lot of interferential bright spots in urban roadway environment, especially in urban arterial roads.

We use the video data captured in an urban roadway environment to verify the reliability and robustness of the proposed algorithm. The videos are segmented into 14 video clips, and each of them contains at least one vehicle. Many of the clips contain multiple vehicles in multi-lane scenarios. The ground truth of total number of vehicles in video segments is drawn by human observation. Then, Table 1 outlines the results of the vehicle detection algorithm from this video data. In Table 1, “Total number” refers to all of the proceeding vehicles in the video frame; “Detection rate” means the ratio of the number of the correctly detected vehicles to the total number of vehicles; “False-negative rate” represents the ratio of the number of false negative detections to the total number of vehicles; and “False-positive rate” means the ratio of the number of detected vehicles which are the false positive detection to the total number of vehicles. From Table 1, we observe that the proposed algorithm could provide a relatively excellent performance.

To demonstrate the superiority of the proposed algorithm, we first explain why the proposed algorithm could avoid the false positive detections. The comparison between the proposed algorithm and the classical global algorithm is presented. In multi-lane urban roads or motorway environments, the false positive detections are usually encountered because of the actual taillights being paired with disturbing bright spots such as street lamps, reflecting marks, *etc.* As the proposed algorithm only detects the pair of the taillights in the PR, thus any false pairing between the spot in the PR and those out of the PR can be avoided.

**Figure 7.**
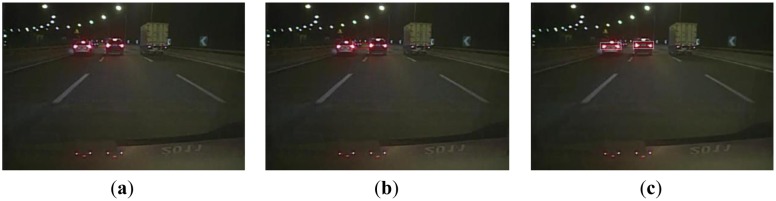
(**a**) A scene with multiple cars driving side-by-side; (**b**) Using the global detection algorithm; (**c**) Using the proposed algorithm.

Figure 7 displays a scene of multiple preceding vehicles at almost the same distances from the host vehicle in the image. The features of these different preceding vehicles are similar and satisfy all the detection rules. As the taillights of the adjacent vehicles in the left have similar location, size, color, shape and symmetry features, this leads to a false detection, as shown in Figure 7b. However, the proposed algorithm could eliminate this false detection, as shown in Figure 7c.

**Figure 8.**
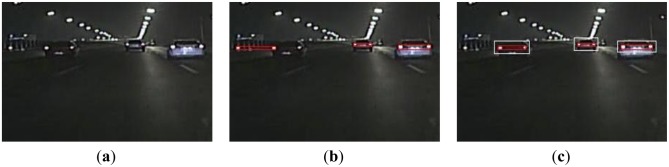
(**a**) A scene with multiple disturbed bright spots; (**b**) Using the global detection algorithm; (**c**) Using the proposed algorithm.

For the scene with multiple disturbed bright spots, we show the results in Figure 8. As the disturbed bright spot is similar to the left taillight of the preceding vehicle in the left, it results in a false detection, as shown in Figure 8b. However, the proposed algorithm could obtain the right detection, as shown in Figure 8c. Overall, the above experiment results indicate that the proposed method can effectively reduce the false detection rate.

**Figure 9.**
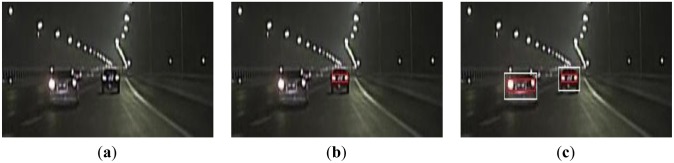
(**a**) A scene when the steering lamp of a preceeding vehicle is working; (**b**) Using the global detection algorithm; (**c**) Using the proposed algorithm.

**Figure 10.**
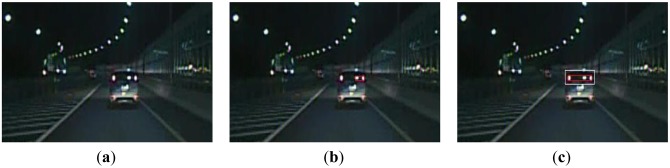
(**a**) A scene where a reflective light block is in the middle of a pair of taillights; (**b**) Using the global detection algorithm; (**c**) Using the proposed algorithm.

**Figure 11.**
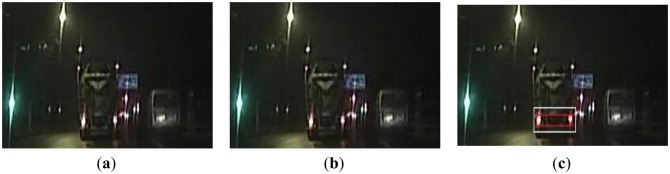
(**a**) A scene where taillights are distorted due to the vibrations of the host vehicle; (**b**) Using the global detection algorithm; (**c**) Using the proposed algorithm.

In comparison to the existing vehicle detection algorithms, another advantage of the proposed algorithm is the adaptive thresholds utilized in the rules of taillights extraction and pairing. The thresholds are adapted in real time according to the corresponding vehicle information in the previous frame. This mechanism could effectively reduce the false positive and false negative detections, as shown in the traffic scenes of Figures 9, 10 and 11.

Figure 9 shows a scene where the symmetry of a pair of taillights being broken when the left steering lamp is working. The existing global detection algorithm would cause a false negative detection, as shown in Figure 9b. As the symmetry threshold is adapted real-time in the proposed algorithm, it is adaptive to the traffic scene changes. Thus, the taillights can still be paired accurately. Figure 10 describes a scene where a reflective light block is in the middle of a pair of taillights, and the light spot is strikingly similar to the right taillight. A false detection occurs due to the global detection algorithm in this case, as shown in Figure 10b. However, the proposed algorithm obtains the correct result, as shown in Figure 10c. For the scene where the taillights are distorted because of the vibrations of the host vehicle, the detection results by the two algorithms are shown in Figure 11. Due to the vibrations of the host vehicle, the similarity of taillights does not satisfy the rule of the global algorithm, thus a false negative detection occurs. By utilizing the adaptive thresholds, the proposed algorithm could derive the right result, as shown in Figure 11c. In summary, the adaptive thresholds based on the tracked PR make the detection adapt to the practical traffic scenes, and thus the false positive and false negative detection rates can be reduced simultaneously.

In the following, we present a comparison between the proposed algorithm and the global algorithm which adds the tracking method. The Kalman filtering is a least-squares estimator of linear movements, which is often applied for preceding vehicle detection in the literature. O'Malley *et al.* [7,8] used the Kalman filtering method to track the location of the taillights by the previous locations and verified the effectiveness of the tracking algorithm through a large number of experiments. The tracking strategy presented by O'Malley *et al.* [8] is summarized as follows: first, a prediction stage estimates the position of tracked targets. After the vehicle detection process, a correction stage associates the detections with tracked targets. These detections are used to update the trackers by the Kalman filtering update equations. If the target fails to be detected and the associated tracker fails to update, the position predicted by the tracking system is to be evaluated. The correlation coefficient of the candidate target and the corresponding region in previous frame is calculated, and compared with the threshold value (0.85).

For simplicity, the algorithm proposed in this paper, the global algorithm, and the Kalman tracking based global algorithm [3,18,22,23] are referred to as *M*_1_, *M*_2_, and *M*_3_, respectively. Comparisons between the three detection algorithms are performed through the video clips a-d. Video clips a-b are captured on a main road with complicated traffic environments, while video clips c-d are captured on an urban expressway with smaller interferential bright spots. The frames of the four videos are 870, 899, 899 and 1,259, respectively. More details of the experiments about the four videos are given in the online Supplementary Material.

Tables 2, 3 and 4 show the results of the three algorithms regarding the detection rate, false positive detection rate and false negative detection rate, respectively. The comparison results show that the proposed algorithm obtains the highest accuracy, *i.e.*, the highest detection rate, the lowest false positive detection rate, and the lowest false negative detection rate. As the tracking algorithm is added in *M*_3_, it has a low false negative detection rate. However, there is no significant increase in the false positive detection rate. Under some complicated traffic conditions, the false positive detection rate of *M*_3_ is even higher than the global detection algorithm, e.g., video b and video c. This implies that the classical combination of the global algorithm and the tracking algorithm could only reduce the false negative detection rate and it has little impact on the false positive detection rate. The reason is that the tracking mechanism does not change the taillights' pairing rules. However, the proposed algorithm could solve this problem.

To conduct a fair comparison of the computing times, all three algorithms are implemented on the same platform. The average computing times of a single frame by the three algorithms are 0.142, 0.201, and 0.227 s, respectively. Obviously, the proposed algorithm derives the shortest computation time, while the computation time of *M*_3_ is the longest of the three algorithms. The reason is that the tracking method takes up some computing time. Moreover, in our proposed algorithm, the average computing time of the three main steps, *i.e.*, image segmentation, PRs predicting, and detection in PRs, are 0.0218, 0.0014, and 0.0236 s, respectively. This indicates that the computing time for the detection in PRs is much smaller than that with the global algorithm. Therefore, the proposed algorithm could improve the detection accuracy and reduce the detection time simultaneously, and thus provide a better nighttime vehicle detection performance than other existing methods.

## 5. Conclusions

In this paper, we study the problem of detecting the preceding vehicles in nighttime traffic scenes via the image processing technique. Based on the brightness of the taillights at nighttime, we use the features, e.g., length-width ratio, intensity, shape, color, symmetry, to identify the taillight pairs at night. To improve the detection accuracy, we propose a region tracking-based vehicle detection algorithm to detect the pairs of the taillights. First, the global detection is used to detect the vehicles. When the vehicle is detected, it is tracked by the AR model. Then, a PR is determined based on the predicted position by the AR model. The vehicle in the next frame is detected in the PR by an adaptive algorithm proposed in this paper. Therefore, the detection time is reduced and the false pairings between bright spots in the PR and the bright spots out of the PR are eliminated. Moreover, when the vehicles are undetected, the predicted position is also used to compensate for the unavailable detection. The usefulness and robustness of the proposed model is demonstrated via some practical videos during nighttime. By comparing the proposed algorithm with the existing classical algorithm, it is shown that the proposed algorithm could simultaneously reduce the false negative detection rate and the false positive detection rate with less detection time. Experimental results show that the total detection rate of the proposed algorithm reaches 97.472%.

Although the experimental studies show that our algorithm is a simple but efficient algorithm for the preceding vehicle detection, there are several issues that need to be further studied. Firstly, the algorithm should be implemented on a hardware platform. Secondly, there are usually some false negative detections caused by reflections of license plate or the rear of the vehicle, and this issue needs to be resolved in the future work. Lastly, the video of this paper is derived through a CCD camera, which has a limited dynamic range. Due to the large variations of scene radiance, this limitation could result in over-exposed or under-exposed conditions. Using a High Dynamic Range (HDR) sensor to solve this problem is another future task regarding our research.

## Figures and Tables

**Table 1.** Vehicle detection results summary.

**Frames**	**Total Number**	**Detection Rate (%)**	**False-Negative Rate (%)**	**False-Positive Rate (%)**
19,745	22,072	97.472	1.314	1.264

**Table 2.** Comparisons regarding the detection rate.

**Detection Rate (%)**	***M*_1_**	***M*_2_**	***M*_3_**
a	100	93.79	95.17
b	100	92.55	96.77
c	98.67	59.89	66.98
d	93.45	73.52	84.24

**Table 3.** Comparisons regarding the false positive detection rate.

**False Positive Detection Rate (%)**	***M*_1_**	***M*_2_**	***M*_3_**
a	0	0.34	0.34
b	0	3.23	4.23
c	1.11	1.77	2.49
d	3.04	17.94	15.19

**Table 4.** Comparisons regarding the false negative detection rate.

**False Negative Detection Rate (%)**	***M*_1_**	***M*_2_**	***M*_3_**
a	0	6.21	4.83
b	0	7.45	3.23
c	1.33	40.11	33.02
d	6.55	26.48	16.71
